# Commentary: The Development of Gonadotropins for Clinical Use in the Treatment of Infertility

**DOI:** 10.3389/fendo.2020.00151

**Published:** 2020-03-20

**Authors:** Fabiola Beligotti

**Affiliations:** Reproductive Medicine and Maternal Health, Ferring Pharmaceuticals, Saint-Prex, Switzerland

**Keywords:** human menopausal gonadotrophin (hMG), assisted reproductive technologies (ART), ovarian stimulation, follicle stimulating hormone (FSH), follitropin delta

Almost a century has passed since the development of the first commercially available gonadotropin. With their recent article, entitled “*The Development of Gonadotropins for Clinical Use in the Treatment of Infertility,”* Lunenfeld et al. provide an overview of the major milestones in the development of gonadotropin products. The authors also describe the issues that affected the decision making during the development processes, and summarize the available evidence supporting the use of recombinant gonadotropin products for the treatment of infertility. While this review provides an insightful overview of the proposed topics, we feel that some of the content would benefit from additional clarification to ensure that the article provides a fair and balanced review of available data.

The review begins by highlighting what Lunenfeld et al. believe to be the major events in the development of gonadotropins, which are then depicted in Figure 1. While the authors rightly dedicate a full sub-section of the article to data on follitropin delta, which was approved by European Medicines Agency (EMA) in 2016, we noticed that this milestone has been omitted from Figure 1. We feel that its inclusion in the figure would grant a more complete and accurate representation of the milestones in gonadotropin development.

Following an overview of available recombinant gonadotropins such as follicle-stimulating hormone, follitropin alfa, and follitropin beta, the authors delineate existing data on follitropin delta. The ESTHER-1 trial is fittingly included and described, as it remains one of the primary randomized clinical trials demonstrating the non-inferiority of follitropin delta to follitropin alfa for the co-primary endpoints of ongoing pregnancy rate and implantation rate. However, a typographical error was made in the description of the dosing regimen tested in the ESTHER-1 trial: it should state that the starting dose was individualized based on body weight and anti-Müllerian hormone, and not body mass index. We feel that this should be corrected to avoid any potential confusion with healthcare physicians who may prescribe this gonadotropin (1).

Furthermore, in the section that discusses the European public assessment reports (EPAR) for follitropin delta, published by the EMA, we noticed that Lunenfeld et al. cite comments posted on an online discussion board for the *Fertility and Sterility* journal (references 75 and 76). These citations were used to support the premise that non-inferiority was not demonstrated for women aged ≤37, and that the dose in the follitropin delta arm was individualized according to clinical markers while the dose of follitropin alfa was at the lower end of the recommended range, therefore reducing the comparability of the outcomes. All queries related to the ESTHER-1 trial, including those discussed in this review, were addressed to the satisfaction of the EMA, leading to the European approval of the product. Citing the EPAR directly would provide the readers with a more complete understanding of the EMA discussions related to the non-inferiority conclusions of the ESTHER-1 trial (2).

The review correctly highlights that follitropin alfa and delta have different pharmacological properties *in vitro*, and that these could contribute to the different properties observed in women. However, it states that follitropin delta has a more rapid clearance compared with follitropin alfa in rats, resulting in lower apparent potency. It should be clarified that the lower potency cited has actually only been observed as an apparent potency in the Steelman–Pohley bioassay in rats. The cited study also explained that when dosing by IU, a unit derived from activity in rats, an equivalent dose of follitropin delta would be expected to result in higher exposure and activity compared with an equivalent dose of follitropin alfa in patients, and therefore, as indicated by Lunenfeld et al. follitropin delta cannot be dosed in the same way as follitropin alfa (3).

One of the last sections of the review is dedicated to the luteinizing hormone and provides an overview of data from studies to date. Lunenfeld et al. rightfully include the ESPART trial, as it was one of the primary randomized clinical trials assessing the efficacy of the rhFSH/rhLH combination compared with rhFSH. It remains, nonetheless, important to emphasize that the ESPART trial was a superiority study that failed to meet its primary endpoint. All ensuing *post-hoc* analyses were therefore put in this context (4, 5).

Overall, Lunenfeld et al. have delivered a comprehensive review that will be invaluable in guiding the readers through the main events in the history of gonadotropin development. We hope that our clarifications will provide additional insights that will support the readers' understanding of the review.

## Author Contributions

FB made substantial contributions to the conception and design of the general commentary. She was also responsible for the interpretation of the data included. The author read and approved the final manuscript.

### Conflict of Interest

The author declares that follitropin delta is manufactured by Ferring Pharmaceuticals. The funder, Ferring Pharmaceuticals, inputted in to the writing of this commentary and the author, FB is an employee of Ferring Pharmaceuticals.
